# Editorial: Chronic Liver Disease: New Targets and New Mechanisms

**DOI:** 10.3389/fmolb.2022.963630

**Published:** 2022-07-18

**Authors:** Yanting Ye, Hua Wang, Jinhang Gao, Enis Kostallari

**Affiliations:** ^1^ Lab of Gastroenterology and Hepatology, State Key Laboratory of Biotherapy, West China Hospital, Sichuan University, Chengdu, China; ^2^ Department of Oncology, The First Affiliated Hospital of Anhui Medical University, Hefei, China; ^3^ Division of Gastroenterology and Hepatology, Mayo Clinic, Rochester, MN, United States

**Keywords:** chronic liver disease, NASH, ALD, cirrhosis, hepatitis, biomarkers, therapeutic targets

## Introduction

Chronic liver diseases (CLD), including non-alcoholic fatty liver disease (NAFLD), chronic hepatitis B (CHB), alcoholic liver disease (ALD), affect 800 million people globally and cause 2 million deaths per year (Asrani et al., 2019; Xiao et al., 2019; Kochanek et al., 2020; Cheemerla and Balakrishnan, 2021; Kostallari et al., 2021). Without effective treatment, CLD is likely to progress to liver cirrhosis and hepatocellular carcinoma (HCC), accounting for 3.5% of worldwide deaths (Moon et al., 2020). Although significant efforts have been made to prevent CLD, the morbidity and mortality remain high. It is an unmet need to explore the mechanisms and novel therapeutic strategies to treat liver disease. This Research Topic presents the most recent advances in CLD, including novel molecular and cellular mechanisms, promising therapeutic targets, new drug delivery methods, and biomarker discovery for liver fibrosis.

## New Targets in Non-Alcoholic Fatty Liver Disease and Non-Alcoholic Steatohepatitis

NAFLD, which affects 25% of the global population, is mainly characterized by hepatic steatosis (Younossi et al., 2016). About 10%–15% of NAFLD patients develop a severe form of NAFLD called NASH, which is characterized by increased inflammation and hepatocyte injury (Zhang et al.; Torres et al.). Although NAFLD represents the highest prevalent type of CLD (Younossi et al., 2019), its pathogenesis has not been fully understood. It was reported that inflammasomes, including the NOD-like receptor protein 3 (NLRP3), are linked to the pathophysiology of NASH (Gan et al., 2022). Torres et al. demonstrated that NLRP3 blockage by its antagonist IFM-514 decreased inflammation and fibrosis in a murine model of NASH, suggesting that NLRP3 inhibition may be an attractive therapeutic approach for NASH patients. Several other pathways were also reported as potential therapeutic targets for NAFLD. Since NAFLD is the result of the abnormal accumulation of lipids, understanding the mechanisms of excessive biogenesis of lipids is of high interest. Hydroxysteroid 17β-dehydrogenase 13 (HSD17B13), a newly identified hepatocyte-specific lipid droplet-associated protein, promotes hepatic lipogenesis (Su et al., 2014). Previous studies revealed that HSD17B13 was increased in the liver of NAFLD patients. However, HSD17B13 gene knockout failed to protect the liver from steatosis. Although murine models produced inconsistent results, human genetic surveys uncovered that loss-of-function human HSD17B13 variants are associated with decreased severity of NAFLD/NASH (Zhang et al.). Consistently, clinical trials showed that downregulation of HSD17B13 with RNA interference (RNAi) therapeutic approaches or the selective HSD17B13 inhibitor INI-678 reduced serum ALT and AST levels and fibrosis markers (NCT04565717, NCT04202354 and https://inipharm.com/), suggesting that this strategy presents a great therapeutic potential. Lipid homeostasis is also regulated by the endoplasmic reticulum (ER)-related degradative signaling pathway, which is necessary to eliminate misfolded proteins, limit ER stress, and maintain cell activity (Maiers et al., 2017; Liu et al., 2021). Dysfunction of ER-related degradative signaling pathway influences the metabolism of hepatocytes and biosynthesis of cholesterol in NAFLD (Duwaerts and Maiers).

The Notch signaling pathway is crucial in regulating cell differentiation, proliferation, and apoptosis. Xu and Wang summarized the role of the Notch signaling pathway in hepatic lipid accumulation, insulin resistance, oxidative stress, fibrogenesis, and autophagy progression in NAFLD. In addition to inflammation as one of the main features of NASH, biliary senescence and senescence-associated secretory phenotype (SASP) were also identified as significant contributors to the progress of NAFLD and NASH *via* the recruitment of immune cells (Meadows et al.). Interestingly, in regards to inter-organ communication, liver-eye cross-talk was found to play a possible role in NAFLD progression and diabetic retinopathy was considered a risk factor for HCC (Yuan et al.). Further studies are needed to transfer the above knowledge into clinical application.

## New Marker and Therapeutic Targets in Chronic Hepatitis B

CHB patients tend to develop liver fibrosis with subsequent cirrhosis and HCC. Thus, accurately assessing the stage of CHB is important for clinical management. Xu et al. introduced leukocyte cell-derived chemotaxin 2 (LECT2) as a novel biomarker of liver fibrosis for CHB patients. LECT2 is secreted by hepatocytes and is present in both, liver tissue and serum. LECT2 levels were significantly increased in patients with CHB or liver cirrhosis, and further enhanced with disease severity, suggesting that LECT2 may be a direct and reliable biomarker for CHB. Although the progress in staging cirrhosis is helpful for clinical decision-making, the current therapies for HBV infection are not sufficient to fully eliminate the virus and restore normal immunity. To this end, Zhong et al. discussed the role of cytokines and chemokines in HBV infection and revealed new potential candidates to be considered for immunotherapies. In summary, the novel staging marker for CHB patients and innovative immunotherapies approaches have the potential to improve the clinical management of CHB patients.

## ER-Related Degradative Signaling Pathways in Alcoholic-Associated Liver Disease (ALD)

ALD is an increasing CLD worldwide with high morbidity and mortality (Sehrawat et al., 2020). The uncertainty of ALD pathogenesis makes it difficult to control the disease (Yin et al., 2022). ER contains the majority of the machinery required for xenobiotic detoxification and is important for cellular homeostasis. It is reported that ER-associated degradation (ERAD) plays a protective role in response to alcohol through promoting cytochrome p450 enzyme E1 (CYP2E1) turnover (Duwaerts and Maiers). It is worth mentioning that ERAD also contributes to remit ER stress of hepatocytes in α1-antitrypsin deficiency (Duwaerts and Maiers). Further studies are needed to better understand ERAD and develop novel protective strategies.

## Portal Fibroblasts and Biliary Senescence in Cholestatic Liver Disease

Cholestatic liver disease is one of the most common liver disorders associated with inadequate bile flow concomitant with a noxious bile acid accumulation in the liver and/or systemic circulation (Gijbels et al., 2021). Myofibroblasts, including activated hepatic stellate cells (HSCs) and portal fibroblasts (PFs), are the major source of extracellular matrix in the injured liver to drive liver fibrosis (Lan et al., 2022). Unlike toxic liver injury, cholestatic liver injury activates not only HSCs but also PFs. PFs are activated and play a dominant role in the early stage of liver injury (Lan et al., 2022); however, due to technical difficulties in isolating PFs, the contributions of PFs to liver fibrosis remain only partially understood. Fuji et al. commented on the role of mesothelin (Msln), mucin 16 (Muc16), and thymocyte differentiation antigen 1 (Thy-1) in PFs activation during cholestatic liver fibrosis. They suggested that therapies targeting Msln or PFs may be promising therapeutic strategies for cholestatic liver diseases. Biliary secretory functions of cholangiocytes regulate liver inflammation and fibrosis through the secretion of various molecules. Biliary senescence and senescence-associated secretory phenotype (SASP) were reported to play a pro-inflammatory role and contribute to the progression of biliary diseases, including primary biliary cholangitis (PBC), primary sclerosing cholangitis (PSC), biliary atresia, as well as ALD (Meadows et al.). Thus developing strategies to target PFs and biliary senescence would present a high therapeutic potential.

## New Mechanisms, Targets and Therapeutic Approaches in Liver Cirrhosis

Liver cirrhosis is the end stage of CLD, which is featured by irreversible extracellular matrix deposition and damage of liver structure (Cai et al., 2021). Cellular cross-talks, signals from the microenvironment, as well as intracellular signaling are crucial in the development of liver fibrosis and cirrhosis (Kostallari et al., 2018; Gao et al., 2020; Gao et al., 2021; Zeng et al., 2021). Two reviews in this issue stated how the major types of liver cells, including HSCs, hepatocytes, liver sinusoidal endothelial cells, PFs, cholangiocytes and inflammatory cells, participate in the pathogenesis and development of liver cirrhosis. The relevant signaling pathways that contribute to liver fibrosis and prospective therapeutic targets were described thoroughly (Zhang et al.; Gu et al.). Additionally, Li et al. emphasized the influence of mitochondria dysfunction and hypoxia inducible factor-1α (HIF1α)-induced oxygen imbalance on metabolism and immunity in liver fibrosis.

In addition to developing new drugs, exploring the anti-fibrotic capacity of existing medicines targeting CLD is also interesting. The widely used anti-HBV infection drug tenofovir disoproxil fumarate (TDF) was found to alleviate liver fibrosis *via* its direct antiviral-independent effects; however, the mechanism involved in reducing fibrosis has not been elucidated, yet. Duan et al. applied genomics analysis to prove that TDF may ameliorate CLD by affecting the expression of genes involved in hepatic immune response and metabolic processes *via* mmu-miR-155-5p-NF-κB signaling.

Each liver cell type might respond in a different way to a given drug; thus, targeting a signaling pathway in a specific cell type would be more effective. The review from Gu et al. discussed the recent nano-delivery approaches specific-targeting HSCs, immune cells, hepatocytes, and liver sinusoidal endothelial cells for liver fibrosis. The nanoparticles (NPs), including metal NPs, lipid NPs, polymer NPs, and protein NPs, with controllable size, shape, diverse components, and modifiable surface characteristics, can reduce drug adverse effects meanwhile improve therapeutic effects. However, the efficacy, quality, safety, and cost-effectiveness of NPs need further research.

## Conclusion and Perspectives

The Research Topic encompasses articles spanning from new clinical and basic research findings to reviews that summarize the recent advancements in the mechanisms of CLD. The role of hepatic myofibroblasts, immune cells, sinusoidal endothelial cells, and related signaling pathways involved in inflammation, immunity, and metabolism in CLD is of great interest. Among the numerous potential therapeutic targets for CLD, treatments targeting HSD17B13 achieved encouraging preliminary results in clinical studies. Meanwhile, the present anti-fibrotic agents are neither liver nor fibrosis specific, leading to insufficient efficacy and several side effects. Therefore, seeking a more specific and effective therapeutic strategies is of great interest and presents an urgent need. Fortunately, with the development of nano-technology, NPs show advantages in targeted drug delivery, combination therapy, and theranostics. Potential anti-fibrotic targets combined with nano-technology might bring a new perspective to future therapies. Up to date, the heat shock protein 47 siRNA delivery through lipid NP to HSC is the only NP-based strategy in the clinical stage for the treatment of liver fibrosis (Gu et al.). Further research is needed to explore more appropriate and reliable nano-delivery approaches and integrate them with the novel therapeutic targets.
